# Blood sugar variation during the first 48 hours of hospitalization for patients with sepsis was associated with in-hospital mortality

**DOI:** 10.1186/cc14048

**Published:** 2014-12-03

**Authors:** K-F Chen, C-C Wu

**Affiliations:** 1 Chang Gung University, Taoyuan, Taiwan and Chang Gung Memorial Hospital at Keelung and Linkou, Taiwan

## Introduction

Blood sugar control for patients with sepsis remains controversial. We aimed to test the hypothesis that the variation of blood sugar level is associated with patient outcome in this study.

## Methods

A retrospective cohort study on nontraumatic adult patients who visited the ED of a tertiary hospital in 2010 and had a clinical diagnosis of severe sepsis was conducted. Patients with two sets of blood culture ordered by emergency physicians and at least two blood sugar tests results available during the first 48 hours of hospitalization were included. The coefficients of variation (CoV, the ratio of the standard deviation to the mean) of the blood sugar level were analyzed with multivariate logistic regression models to test the association between in-hospital mortality.

## Results

**Figure 1:**
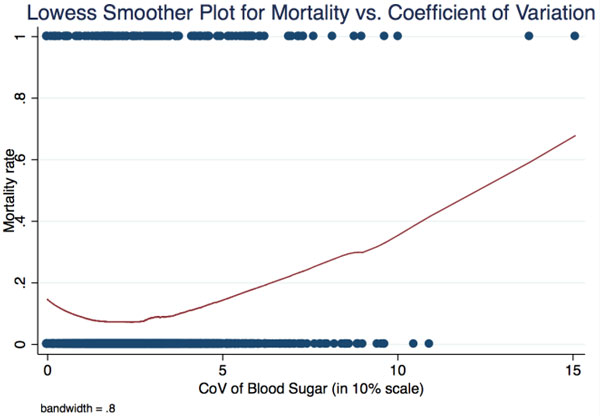
**Lowess smoother plot for mortality versus coefficient of variation**.

Of the 1,537 patients included, most were older than 70 years of age (median; 71, IQR: 59 to 80), male (54%), without a diagnosis of severe sepsis (63%) and had a previous diagnosis of diabetes (84%). The initial blood sugar levels of patients with and without previously diagnosed diabetes were 259 ± 9.9 and 154 ± 5.7, respectively (mean ± SEM). The CoV of the consecutively monitored blood sugar level during the first 48 hours of admission for patients with and without previously diagnosed diabetes were 29.4 ± 0.5% and 21.0 ± 0.5%, respectively. Patients with CoV lower than 10% and higher than 30% tended to have higher mortality rate, compared to patients with 10 to 30% CoV level (11% vs. 12% and 7%, respectively, Figure 1). In the multivariate logistic regression model adjusting for age, initial blood sugar level, severity of sepsis, previous diagnosis of diabetes and diagnosis of severe sepsis, the higher CoV level (>30%) was found to be associated with 60% increased odds of in-hospital mortality (aOR: 1.61 ± 0.34); while the previous diagnosis of diabetes was found to be associated with 45% lower odds of in-hospital mortality (aOR: 0.55 ± 0.13, Table 1).

**Table 1:** 

	Odds ratio	Standard error	*P *value	95% CI	
Sugar CoV <10%	1.51	0.44	0.15	0.86	2.67
Sugar CoV >30%	1.61	0.34	0.02	1.06	2.43
Initial sugar level <100	0.98	0.01	0.00	0.97	0.99
Initial sugar level ≥ 100 and <500	1.00	0.00	0.67	1.00	1.00
Initial sugar level higher ≥ 500	1.00	0.00	0.54	1.00	1.00
Severe sepsis	2.43	0.48	0.00	1.65	3.56
Previous diagnosis of diabetes	0.55	0.13	0.01	0.34	0.88
Severity of sepsis	1.14	0.04	0.00	1.07	1.21
Age	1.01	0.01	0.49	0.99	1.02

## Conclusion

In this retrospectively cohort study, we found that increased blood sugar variation was associated with worse patient outcome. However, further study is merited to test the possible causal relationship between variation of blood sugar level and patient outcome.

